# Care of the Baby

**Published:** 1893-04

**Authors:** 


### CARE OF THE BABY.

A healthy infant will take water every hour, and be the better for it. The less rocking, tossing, patting, combing, coaxing, teasing, and kissing an infant is obliged to endure, the better his health and good nature. See that he sleeps in a cool room, with mouth shut and head uncovered. If you wish to rest at nights think how you would swelter between two giants, and do not put the baby to bed with two grown people. Have all garments loose enough for comfort at throat, arms, waist, and wrists, and be sure to have the shoes and stockings large enough. A child should not be given meat until he is two years old. Do not try to teach a child to stand. He will stand by himself when his body and bones are in condition. Use no starch on his clothing, and keep his bibs dry.

